# Predict the Effects of Dolutegravir (DTG) Plus Lamivudine (3TC) on Immunological Responses in People Living with HIV (PLWHIV)

**DOI:** 10.3390/jcm12031176

**Published:** 2023-02-01

**Authors:** Jesús Troya, Roberto Pedrero-Tomé, Luis Buzón, Carlos Dueñas, on behalf of the SPADE Study Group

**Affiliations:** 1 Department of Internal Medicine, Infanta Leonor University Hospital, 28031 Madrid, Spain; 2 Infanta Leonor University Hospital Research and Innovation Foundation, 28031 Madrid, Spain; 3 EPINUT Research Group, Faculty of Medicine, Complutense University of Madrid, 28040 Madrid, Spain; 4 Department of Internal Medicine, Infectious Diseases Division, Burgos Hospital, 09006 Burgos, Spain; 5 Valladolid University Hospital, Faculty of Medicine, University of Valladolid, 47003 Valladolid, Spain

**Keywords:** HIV, immune recovery, 2DR, dolutegravir, lamivudine, nomograms

## Abstract

Background: Immune recovery in people living with HIV (PLWHIV) is a residual aspect of antiretroviral treatment (ART) in most patients, but in a non-negligible proportion of them, the CD4+ lymphocytes count, or CD4/CD8 ratio remains suboptimal. Methods: We performed a model of the immune response after 24 weeks of switching to a 2DR with DTG plus 3TC in a retrospective multicenter cohort of undetectable and experienced patients using significant predictor variables associated with the parameters or situations defined as success and failure. Clinical variables studied were CD4+ and CD8+ lymphocyte count, percentage of CD4, and CD4/CD8 ratio. These parameters were assessed at baseline and 24 weeks after the switch. Based on the evolution of each variable, four categories of immune response and four categories of non-immune response were defined. Immune response was defined as CD4+ count > 500 cells/mm^3^, %CD4 > 30%, CD8+ count < 1000 cells/mm^3^ and CD4/CD8 ratio ≥ 0.9. Non-response is just the opposite. Results: In our different models of immunological response, the presence of stage of AIDS (*p* = 0.035, *p* = 0.065) and current age over 50 years (*p* = 0.045) are postulated as statistically significative limiting factors in achieving an improvement in CD4, %CD4, CD8, and CD4/CD8 ratio. Late HIV diagnosis (*p* = 0.156), without statistical significance, enhanced late the previous variables. In contrast, conditions where patients start with CD4 > 500 cells/mm^3^ (*p* = 0.054); CD4 > 30% (*p* = 0.054, *p* = 0.084); CD8 < 1000 cells/mm^3^ (*p* = 0.018), and CD4/CD8 ≥ 0.9 (*p* = 0.013, *p* = 0.09) are detected as stimulating or conducive to DTG plus 3TC treatment success. Conclusion: These models represent a proof of concept that could become a valuable tool for clinicians to predict the effects of DTG plus 3TC on immunological responses prior to the switch in undetectable pre-treated PLWHIV with immune dysfunction. The main predictors for immunological failure were late HIV diagnosis, stage of AIDS, and current age over 50 years. In contrast, starting with a normalized immune status was detected as stimulating or conducive to DTG plus 3TC treatment success.

## 1. Introduction

Currently, immune recovery in people living with HIV (PLWHIV) is a residual aspect of antiretroviral treatment (ART) in most of them. This aspect is explained mainly due to the availability of highly potent, effective, and safe therapeutic strategies for naïve [1,2] and pre-treated patients [3,4,5] and, on the other hand, the early initiation of treatment after diagnosis of HIV infection. Nevertheless, approximately 20% of PLWHIV do not achieve immune reconstitution, including suboptimal CD4+ lymphocyte count or CD4/CD8 ratio, despite suppression of viral replication [6,7]. Traditionally, the non-immune response is defined as CD4+ cell counts <200 cells/mm^3^ or less than a 20% increase from baseline. A low CD4/CD8 ratio indirectly represents immune system dysregulation and hyperinflammation. In addition, they are associated with innate and adaptive immune activation and immunosenescent phenotype [8]. On the other hand, an adequate immune response correlates with a CD4+ lymphocyte count of over 500 cells/mm^3^ and a CD4/CD8 ratio over 0.8 [9,10].

The reasons for the poor immunological response are only partially known. They include genetic, demographic, and immunologic factors. Among others, identified factors are late HIV diagnosis with less than 350 cells/mm^3^ [11], delayed start of treatment [12], immunosenescence in older age, viral coinfections [13,14], and bone marrow and thymus dysfunction. The use of additional treatments or highly effective ART to improve immune reconstitution has been investigated, but no conclusive results have been published. The impossibility of recovering immune status after years of effective antiretroviral therapy represents a risky situation for PLWHIV, with increased morbidity and mortality [15,16]. In this sense, a low CD4/CD8 ratio has been associated with an increased risk of non-AIDS-related events and death [17]. Therefore, early HIV diagnosis and initiation of antiretroviral treatment have proven to be the best clinical tools to prevent immune wasting and allow immunological normalization, avoiding the associated increase in morbidity and mortality, as recently demonstrated in the Insight START study [18].

Complete immune restoration is traditionally related to a three-drug regimen (3DR). However, recent studies of dual therapy (2DR) with dolutegravir and lamivudine (DTG + 3TC) versus 3DR have found no apparent differences in CD4/CD8 dynamics as surrogate markers of immune and inflammatory recovery [19,20]. Nevertheless, there is a debate if 2DR, based on integrase inhibitors, mainly DTG + 3TC, is inferior regarding immune and inflammatory recovery [21].

Some PLWHIV remains in a suboptimal immunological state regardless of ART in real-life cohorts. In this situation, the possibility of having complementary and predictive immunological information related to associated factors when choosing a particular ART could be of great interest to clinicians and patients, contributing to more personalized therapy. In this sense, the object of this study is the development of a predicting tool to evaluate who will present immunological improvement based on a 2DR strategy using a retrospective cohort of more than 1000 patients switching to DTG + 3TC.

We performed models of the immune response after 24 weeks of switching to a 2DR with DTG plus 3TC in a retrospective multicenter cohort of undetectable and experienced patients using statistically significant predictor variables associated with the parameters or situations defined as success and failure.

## 2. Material and Methods

### 2.1. Study Population

The study population comprised a multicenter Spanish retrospective cohort of 1032 PLWHIV (811 males; 221 females) treated with DTG plus 3TC at 13 hospitals in Spain. The data were collected from 1 November 2020 to 1 August 2021, in a retrospective multicenter study conducted at 13 hospitals in Spain during the most challenging days of the coronavirus disease 2019 (COVID-19) pandemic. Inclusion criteria comprised pre-treated patients with an undetectable HIV viral load before switching to a 2DR with DTG plus 3TC and a clinical follow-up of at least 24 weeks. Data were obtained from medical records in the 13 hospitals at baseline and weeks 24, 48, and 96. Studied variables included data regarding demographics, HIV infection, comorbidities, immune status (CD4+ and CD8+ lymphocyte count, percentage of CD4, CD4/CD8 ratio), and HIV viral load. Table 1 includes the main demographic and clinical characteristics of the study population.

The study population analysis was only performed at week 24 after the switching to DTG plus 3TC (sample size 1032 patients). Due to the lack of data regarding immunological status in the different models at weeks 48 (samples sizes between 100–120) and 96 (sample size between 65–90), and thus to avoid the limitation of the power of the models, we did not perform further immunological analyses apart from the 24 weeks analysis. The study has been designed in this way to be able to care for patients in a more specific way, taking into account two factors: (1) to predict the evolution of patients with a deficient immune situation and (2) to identify possible factors that lead to reducing the immunity of patients whose baseline immune status is stable.

Bearing that the objectives are reduced to identifying possible prognostic factors of what the authors have defined as success or failure, logistic regression analyses have been performed. In order to further refine the results, those patients who did not show oscillation between baseline values and at 24 weeks had to be discarded. Under ideal conditions, we would have added prediction models at 48 and 96 weeks. However, consistent mathematical models cannot be made with the available data.

Established model pairs offer complementary rather than opposite results. This aspect can be explained because the data used to build each model is different; therefore, the obtained results are not identical but present the same interpretation. For example, the sample size of the model to achieve CD4 ≥ 500 cells/mm^3^ at 24 weeks in PLWHIV to be treated with DTG plus 3TC and with basal CD4 values < 500 cells/mm^3^ is 146 individuals, while that of its complement is 489 (to achieve CD4 < 500 cells/mm^3^ at 24 weeks with basal CD4 values ≥ 500 cells/mm^3^).

### 2.2. Main Objectives

The study aims to develop a model of the immune response after 24 weeks of switching to a 2DR with DTG + 3TC in undetectable and experienced patients using significant predictor variables collected from clinical records in real-life experience.

### 2.3. Definition of the Presence or Not of Immune Response

Immune response was analyzed with CD4+ and CD8+ lymphocyte count (cells/mm^3^), percentage of CD4, and CD4/CD8 ratio. These parameters were assessed at baseline and 24 weeks after treatment. Based on the evolution of each variable, four categories of immune response and four categories of non-immune response were defined to evaluate the effectiveness of the treatment. Immune response was defined as (1) patients who started with CD4 values < 500 cells/mm^3^ and reached CD4 ≥ 500 cells/mm^3^ at 24 weeks; (2) exceeded baseline %CD4 < 30% at 24 weeks; (3) started with baseline CD8 > 1000 cells/mm^3^ and reached lower values at 24 weeks, and (4) started with a baseline CD4/CD8 ≤ 0.9 and reached higher values at 24 weeks. In this regard, it is essential to note that each scenario excludes patients with no oscillation between their baseline and 24-week values. The models were built solely and exclusively, including patients who initially presented a lower basal immunological situation or were cataloged by the authors as deficient (between 30–60% of patients, depending on the model). This selection has been made under the criteria of studying the recovery of those patients with unwanted basal immunological values since it is considered the population group most sensitive to improvement. These definitions were used according to the reference values of normality used in other studies [8,9], although they could have no initial clinical relevance when applied to a particular sample, which is the one proposed in this work. The analysis was not performed at 48 or 96 weeks of treatment due to the small sample size of patients with available data, thus avoiding loss of statistical power.

### 2.4. Variables Included in the Model

The selection of potential predictor variables took into account demographic (gender and age), epidemiological (time of HIV diagnosis and presence of AIDS), clinical (baseline immune status), and other antiretroviral treatment factors. In all models, the following variables were included as inputs: baseline CD4 > 500 cells/mm^3^, % baseline CD4 > 30%, baseline CD8 < 1000 cells/mm^3^, CD4/CD8 ≥ 0.9, time of HIV diagnosis (<5 years; 5–9 years and ≥10 years), patient age ≥ 50 years, presence of associated comorbidities, previous use of INSTI, NNRTI, PI, and the FTC/TDF, FTC/TAF, ABC/3TC backbones. According to the algorithms of the stepwise logistic regression model, all the variables without significant statistical value were sequentially discarded until concluding with the most parsimonious models for each case. The selection of the variables that enter the final model is not the authors’ decision. It depends exclusively on the mathematical algorithms behind the stepwise logistic regression model. In this type of model, it is common to include variables that, despite not being significant (*p* < 0.05), enhance the effect of the other essential variables (*p* < 0.05). The variables that are not included in the model are extracted either because they do not present a significance on the study’s dependent variable or because they do not present a potentiating effect on the rest of the independent variables.

### 2.5. Statistics

The statistical procedure was performed using the R Core Team software (2022) [21]. The descriptive statistics of the patient’s characteristics and possible predictors were presented as frequencies (n) and percentages (%) in the case of categorical variables and the median and interquartile range (IQR) for continuous variables. A χ^2^ test or Fisher’s exact test was applied to assess the differences between groups of categorical variables, and a T-Student or Mann–Whitney U test was used for continuous variables. Eight predictive models were used to define the presence or not of immune response for each immunological parameter using backward stepwise multiple logistic regression analysis. The odds ratio (OR) and associated 95% confidence interval (CI) were obtained for each adjusted multivariate model. In addition, by the R function named the *Hoslem* test, the models were internally evaluated using the Homer–Lemeshow test, which allows for examining whether the observed proportions are similar to the predicted probabilities in subgroups of the data set. We accepted the null hypothesis that the proportions observed in the overall cohort are maintained in random subgroups of the data set. Subsequently, using the R rms package, the results derived from the regression models were plotted on eight nomograms. These models could be complementary two by two, depending on the parameter to be studied, since, for each parameter, there is one model capable of predicting success and one capable of predicting failure in immune recovery.

## 3. Results

In the Spade cohort, the analysis for paired data showed an increase in CD4+ lymphocyte count after switching to a 2DR with DTG plus 3TC at 24 and 48 weeks. No statistically significant differences were observed in the study of paired samples based on gender. However, in patients without a previous diagnosis of AIDS, a significant reduction (*p* < 0.05) was observed in the number of CD8+ lymphocyte count at 48 and 96 weeks [−27.8 (SD: 318.3); −54.0 (SD: 266.1)].

After statistical analysis, the significative predictor variables for this model in terms of positive or negative impact on immunological response and logistic regression models associated with the parameters or situations previously defined as success (models 1–4) and failure (models 5–8) are described in Table 2. Variables associated with immune response include (a) to achieve CD4 ≥ 500 cells/mm^3^: age (*p* = 0.045), time of HIV diagnosis (*p* = 0.156), AIDS diagnosis (p = 0.035), baseline percentage of CD4 over 30% (*p* = 0.083), and previous NNRTI treatment (p = 0.041); (b) to achieve CD4 ≥ 30%: AIDS diagnosis (*p* = 0.065), previous FTC/TDF treatment (p = 0.062), baseline CD4+ over 500 cells/mm^3^ (0.054), and baseline CD4/CD8 ratio over 0.9 (0.013); (c) to achieve CD8 ≤ 1000 cells/mm^3^: comorbidity (*p* = 0.003), previous INSTI treatment (*p* = 0.068), and baseline CD4/CD8 ratio over 0.9 (0.090); (d) to achieve CD4/CD8 ≥ 0.9: baseline CD4 percentage over 30% (*p* = 0.054), CD8+ less than 1000 cells/mm^3^ (*p* = 0.018), and previous treatment with NNRTI (*p* = 0.083).

The level of significance of the variable time of HIV diagnosis (*p* > 0.05) may be striking. However, it is a variable selected by the model algorithm, so it enhances the effects of the rest of the variables.

As indicated by the odds ratios (OR) of the first set of models, late HIV diagnosis, the presence of AIDS, and current age over 50 years are postulated as limiting factors in achieving an improvement in CD4, %CD4, CD8, and CD4/CD8 ratio. In contrast, conditions where patients start with CD4 ≥ 500 cells/mm^3^; CD4 ≥ 30%; CD8 ≤ 1000 cells/mm^3^ and/or CD4/CD8 ≥ 0.9 are detected as stimulating or conducive to DTG plus 3TC treatment success. These data are quite consistent with those obtained for models 5–8. Thus, certain variables (age or presence of AIDS) that limited treatment success now play a prognostic factor for treatment failure. In the same way, the variables previously identified as possible immune system enhancers would develop an effect of containment of failure.

**Figure 1:**
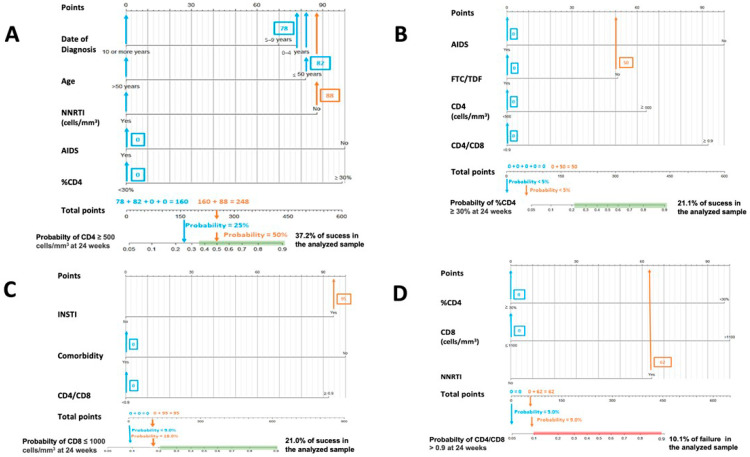
Nomograms to predict the probability of success at 24 weeks. (**A**) Nomogram to predict the probability of achieving CD4 ≥ 500 cells/mm^3^ at 24 weeks in PLWHIV treated with DTG plus 3TC and with basal CD4 values < 500 cells/mm^3^: (**B**) nomogram to predict the probability of achieving CD4 ≥ 30% at 24 weeks in PLWHIV treated with DTG plus 3TC and with basal %CD4 < 30%; (**C**) nomogram to predict the probability of achieving CD8 ≤ 1100 cells/mm^3^ at 24 weeks in PLWHIV t treated with DTG plus 3TC and with basal CD8 > 1100 cells/mm^3^; (**D**) nomogram to predict the probability of achieving CD4/CD8 ≥ 0.9 at 24 weeks in PLWHIV treated with DTG plus 3TC and with basal CD4/CD8 < 0.9.

**Figure 2:**
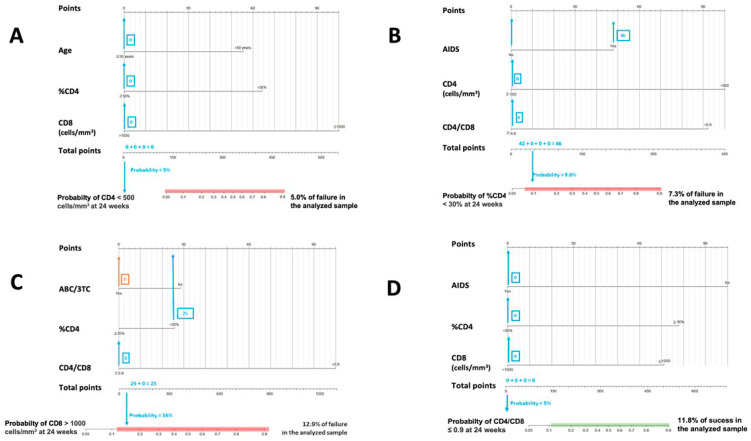
Nomograms to predict the probability of failure at 24 weeks. (**A**) Nomogram to predict the probability of achieving CD4 < 500 cells/mm^3^ at 24 weeks in PLWHIV treated with DTG plus 3TC with basal CD4 values ≥ 500 cells/mm^3^; (**B**) nomogram to predict the probability of achieving CD4 < 30% at 24 weeks in PLWHIV treated with DTG plus 3TC and with basal ≥ %CD4 30%; (**C**) nomogram to predict the probability of achieving CD8 > 1100 cells/mm^3^ at 24 weeks in PLWHIV treated with DTG plus 3TC and with basal CD8 ≤ 1100 cells/mm^3^; (**D**) nomogram to predict the probability of achieving CD4/CD8 ≤ 0.9 at 24 weeks in PLWHIV treated with DTG plus 3TC and with basal CD4/CD8 > 0.9.

Figure 1 and Figure 2 are nomograms, a series of visual representations of this research’s practical and clinical application. This type of graph is offered to simplify the interpretation and usefulness of the models proposed above, allowing the calculation of the probability of a phenomenon (for example, obtaining a CD4+ value higher than 500 cells/mm^3^) through the values presented by certain predictor variables previously selected by the binary stepwise logistic regression models (for example age category, sex, initial CD8+ count value, …).

Figure 1 and Figure 2. In both cases, the calculated probabilities of treatment success and failure of two random patients from the database are exemplified using the proposed nomograms. For the exemplification in Section 1, a 48-year-old male AIDS patient with a baseline CD4+ lymphocyte count of 395 cells/mm^3^, CD4% of 13.6%, CD8+ lymphocyte count of 1641 cells/mm^3^, and a CD4/CD8 ratio = 0.24 was randomly selected. Section 2 calculations are made for a 34-year-old male with a CD4+ lymphocyte count of 1398 cells/mm^3^, a CD8+ lymphocyte count of 1399 cells/mm^3^, and a CD4/CD8 ratio = 0.99.

In Figure 1, the area in which the probability of success would be equal to or greater than that found in the sample is marked in green. Similarly, in Figure 2, the probabilistic area in which the probability of failure is equal to or higher than that detected internally in the present study is marked in red. Thus, ideally, it would be possible to locate the patient within the green band and outside the red zone. In addition, all variables in which the clinician cannot or has not been able to intervene (date of diagnosis, age, presence of AIDS, …) are marked in blue, and those in which intervention is possible, such as the use of other drugs, are marked in orange. Thus, in some instances, up to two probabilities associated with the same patient are offered: one with previous intervention by the clinician (orange) and one without (blue).

The interpretation of these figures is relatively straightforward. To manually obtain the probability of a patient receiving an improvement or failure in one of the parameters studied, we should locate the patient’s baseline characteristics in the specific nomogram. We would then draw a vertical line to the score assigned for the particular variable and add that figure to the other elements to obtain a total score (e.g., in nomogram A in Figure 1, an age at HIV diagnosis of fewer than four years, scores 78). Then, with that number and in the lower “total score” section, a vertical line would be drawn downwards to obtain the associated probability. According to these models, the first patient would not be in the ideal baseline scenario to achieve an improvement classified as a success. In contrast, the second patient appears far from the established cut-off points for estimating CD4+ count and CD4/CD8 failure in patients treated with DTG plus 3TC. However, as the estimated probabilities are slightly elevated, special care should be taken with CD8+ count and percentage of CD4.

## 4. Discussion

To date, no studies using predictive tools to measure the impact of factors related to immune recovery are available, as they only manage to isolate predictive factors for this immune recovery [22]. In this sense, our tool could greatly interest clinicians in patients with immune dysfunction (CD4+ lymphocyte count < 500 cells/mm^3^, CD4/CD8 ratio < 0.9, CD8+ lymphocyte count > 1000 cells/mm^3^), despite virological control of HIV infection with ART, before switching to a 2DR with DTG plus 3TC. On the one hand, our study creates several nomogram models that identify the factors that could significantly contribute to improving or not their immune response [9,10]. On the other hand, it weighs the impact of each analyzed factor on each patient. In this sense, significant variables such as time since diagnosis, age, and the presence of AIDS interfere with immune response, and these failure models could give relevant prognostic information before switching to a 2DR with DTG plus 3TC. Furthermore, published data shows these factors have been widely related to negative mitigating factors for immune recovery [13,14].

In real-life cohorts, approximately 20% of PLWHIV do not achieve immune reconstitution despite suppression of viral replication, even after years of effective antiretroviral therapy [6,7,23]. Suboptimal immune recovery has been clinically related to some factors such as aging, increased innate and adaptive immune activation and immunosenescence phenotype, low nadir CD4+ count below 200 cells/mm^3^, low CD4/CD8 ratio, AIDS, and poor baseline clinical status, among others [8,24,25]. Our study corroborates these data in their positive and negative implications regarding immunological response. In our models, the presence of the AIDS stage and the current age over 50 years, enhanced by late HIV diagnosis, are postulated as limiting factors in achieving an improvement in absolute CD4, CD8 %CD4, and CD4/CD8 ratio. In contrast, conditions where patients start with CD4 ≥ 500 cells/mm^3^; CD4 ≥ 30%; CD8 ≤ 1000 cells/mm^3^ and/or CD4/CD8 ≥ 0.9 are detected as stimulating or conducive to DTG plus 3TC treatment success.

There is a debate on the potential inferiority of a 2DR strategy based on integrase inhibitors concerning a 3DR strategy regarding immune and inflammatory recovery. Some studies have related it to macrophage activation due to a potentially diminished drug pressure [25]. Nevertheless, preliminary results of real-life experience contradict this approach [19,20]. Our findings in the SPADE cohort corroborate real-life cohort results with a significative CD4+ lymphocyte count improvement and a CD8+ lymphocyte normalization at 24 and 48 weeks after switching in those patients with no limiting factors. In addition, our study complements this data by identifying those factors that could influence the success (baseline immune status) or failure (AIDS, current age over 50 years, and time of HIV diagnosis) of immune recovery in the first 24 weeks after switching to a 2DR with DTG plus 3TC.

On the other hand, these models could quantify the percentage of risk or success associated with a patient who presents more than one unfavorable situation, weighing the role of each factor and the potential immunological implications of switching in those patients with limiting factors.

This study has some limitations due to the retrospective nature and the specific characteristics of these PLWHIV switching to DTG plus 3TC. Firstly, these models are performed in a long-term cohort of pre-treated PLWHIV, with more than ten years of evolution and pre-exposed to older ARTs (NNRTIs and b/PIs). This situation could differ from PLWHIV diagnosed in the last five years, with better immune status at diagnosis, earlier initiation of treatment [18] and mostly treated with regimens containing an INSTI. Secondly, the absence of a proper analysis at weeks 48 and 96 due to the small sample size of patients with available data, thus avoiding loss of statistical power analysis. Thirdly, since the models were built only based on the patients with oscillation, the results can only be generalized to those patients with oscillations between baseline and 24 weeks. Finally, another limitation resides in the lack of data concerning the potential effect of other viral coinfections, such as cytomegalovirus, Epstein–Barr or Hepatitis C or B virus.

This study represents a proof of concept in a multicenter retrospective cohort of pre-treated patients switching to DTG plus 3TC, and cross-validation studies with other retrospective or prospective cohorts are mandatory to assess these models’ validity in real-life experience in HIV patients with immune dysfunction. As described in the Section 2, the models were internally evaluated using the Homer–Lemeshow test and the proportions observed in the overall cohort are maintained in random subgroups of the data set. Nevertheless, their clinical application must be cautiously made.

To conclude, these models represent a proof of concept that could become a valuable tool for clinicians to predict the effects of DTG plus 3TC on immunological responses prior to the switch in undetectable pre-treated PLWHIV with immune dysfunction. The main predictors for immunological failure were the stage of AIDS, and the current age over 50 years, enhanced by late HIV diagnosis. In contrast, conditions where patients start with CD4 ≥ 500 cells/mm^3^; CD4 ≥ 30%; CD8 ≤ 1000 cells/mm^3^ and/or CD4/CD8 ≥ 0.9 were detected as stimulating or conducive to DTG plus 3TC treatment success.

## Figures and Tables

**Table 1:** Main characteristics of PLWHIV treated with DTG + 3TC in the SPADE cohort.

	Overall (n = 1032)	Female (n = 221)	Male (n = 811)	*p*-Value	Non-AIDS (n = 634) ^	AIDS (n = 118)	*p*-Value
Demographics							
Age, median (IQR)	10.0 [4.0, 22.0]	11.0 [5.0, 23.0]	10.0 [4.0, 20.0]	<0.07	9.0 [4.0, 20.0]	11.0 [4.0, 22.0]	<0255
Time of HIV diagnosis, median (IQR)	37.0 [27.0, 47.0]	38.0 [29.0, 49.0]	36.0 [26.0, 46.0]	0.126	34.0 [24.0, 45.0]	45.5 [32.0, 53.8]	<0.001
Male, n (%)	811 (78.6)	-	-	<0.001	512 (80.8)	89 (75.4)	0.229
Spanish nationality, n (%)	764 (76.9)	156 (73.2)	608 (77.8)	0.186	405 (66.9)	97 (82.2)	0.001
Comorbidities, n (%)							
Arterial hypertension	119 (11.5)	26 (11.8)	93 (11.5)	0.997	83 (13.1)	34 (28.8)	<0.001
Diabetes	50 (4.8)	9 (4.1)	41 (5.1)	0.67	35 (5.5)	15 (12.7)	0.007
Dyslipidemia	211 (20.4)	48 (21.7)	163 (20.1)	0.663	168 (26.5)	41 (34.7)	0.085
Heart Disease	29 (2.8)	4 (1.8)	25 (3.1)	0.432	19 (3.0)	9 (7.6)	0.030
Cerebrovascular disease	9 (0.9)	2 (0.9)	7 (0.9)	1.000	4 (0.6)	5 (4.2)	0.004
Peripheral vascular disease	11 (1.1)	2 (0.9)	9 (1.1)	1.000	8 (1.3)	3 (2.5)	0.518
Kidney failure	40 (3.9)	6 (2.7)	34 (4.2)	0.417	28 (4.4)	12 (10.2)	0.020
Osteoporosis/osteopenia	31 (3.0)	13 (5.9)	18 (2.2)	0.009	24 (3.8)	7 (5.9)	0.409
Chronic pulmonary disease	48 (4.7)	14 (6.3)	34 (4.2)	0.246	36 (5.7)	11 (9.3)	0.196
Psychiatric disorders	78 (7.6)	23 (10.4)	55 (6.8)	0.096	60 (9.5)	17 (14.4)	0.144
Cancer	14 (1.4)	5 (2.3)	9 (1.1)	0.325	10 (1.6)	4 (3.4)	0.334
Chronic liver disease	106 (10.3)	30 (13.6)	76 (9.4)	0.089	71 (11.2)	35 (29.7)	<0.001
Number of comorbidities, n (%)							
One	617 (59.8)	126 (57.0)	491 (60.5)	0.439	315 (49.7)	26 (22.0)	<0.001
Two	220 (21.3)	45 (20.4)	175 (21.6)		181 (28.5)	37 (31.4)	
Three	105 (10.2)	24 (10.9)	81 (10.0)		79 (12.5)	25 (21.2)	
Four	54 (5.2)	17 (7.7)	37 (4.6)		35 (5.5)	18 (15.3)	
Five	29 (2.8)	8 (3.6)	21 (2.6)		19 (3.0)	10 (8.5)	
Six	4 (0.4)	0 (0.0)	4 (0.5)		4 (0.6)	0 (0.0)	
HIV infection							
Transmission pathways, n (%)							
Sexual intercourse	684 (67.8)	118 (53.6)	566 (71.7)	<0.001	426 (69.4)	64 (54.7)	<0.001
Intravenous drug injectors	191 (18.9)	61 (27.7)	130 (16.5)		84 (13.7)	35 (29.9)	
Immune status, median (IQR)							
Baseline CD4+ (cells/mm^3^)	753.0 [549.0, 977.0]	763.0 [590.5, 985.0]	744.0 [543.0, 975.8]	0.358	786.5 [596.5, 1005.8]	604.0 [404.5, 933.0]	<0.001
24 weeks CD4+ (cells/mm^3^)	770.5 [592.8, 980.0]	785.0 [601.0, 986.5]	766.0 [592.0, 970.8]	0.482	808.0 [630.5, 1000.5]	644.0 [449.0, 875.5]	<0.001
48 weeks CD4+ (cells/mm^3^)	782.0 [574.0, 1004.0]	779.0 [606.0, 978.0]	784.5 [567.0, 1012.5]	0.996	801.0 [591.5, 1029.5]	628.0 [424.2, 866.0]	<0.001
96 weeks CD4+ (cells/mm^3^)	823.0 [613.2, 1048.0]	802.0 [652.0, 1037.0]	839.0 [604.5, 1051.0]	0.854	851.0 [678.0, 1125.0]	649.0 [440.5, 856.2]	<0.001
Baseline CD8+ (cells/mm^3^)	867.5 [630.0, 1179.5]	805.0 [589.5, 1084.5]	878.0 [653.0, 1196.5]	0.048	875.0 [637.5, 1199.5]	827.0 [609.0, 1100.0]	0.141
24 weeks CD8+ (cells/mm^3^)	897.0 [656.0, 1220.0]	792.0 [603.0, 1188.5]	913.0 [674.0, 1247.0]	0.020	899.5 [656.8, 1241.0]	871.0 [635.0, 1134.0]	0.517
48 weeks CD8+ (cells/mm^3^)	908.0 [638.5, 1229.8]	817.0 [533.0, 1119.0]	922.0 [661.5, 1248.5]	0.020	900.0 [635.5, 1220.5]	959.5 [603.2, 1224.8]	0.651
96 weeks CD8+ (cells/mm^3^)	906.0 [628.5, 1241.5]	922.0 [625.0, 1222.5]	906.0 [634.5, 1268.5]	0.903	956.0 [672.0, 1246.0]	862.0 [484.5, 1152.5]	0.094
Baseline CD4+/CD8+ (cells/mm^3^)	0.9 [0.6, 1.2]	0.9 [0.7, 1.4]	0.9 [0.6, 1.2]	0.033	0.9 [0.7, 1.3]	0.8 [0.5, 1.1]	0.003
24 weeks CD4+/CD8+ (cells/mm^3^)	0.9 [0.6, 1.2]	1.0 [0.7, 1.3]	0.8 [0.6, 1.2]	0.011	0.9 [0.7, 1.2]	0.7 [0.5, 1.1]	0.001
48 weeks CD4+/CD8+ (cells/mm^3^)	0.9 [0.6, 1.2]	1.0 [0.7, 1.4]	0.9 [0.6, 1.2]	0.034	0.9 [0.7, 1.3]	0.7 [0.5, 1.0]	<0.001
96 weeks CD4+/CD8+ (cells/mm^3^)	0.9 [0.7, 1.3]	0.9 [0.7, 1.3]	0.9 [0.7, 1.3]	0.770	0.9 [0.7, 1.4]	0.8 [0.7, 1.2]	0.198
HIV diagnosis n (%)							
Previous treatments, n (%) ^^							
ABC/3TC	384 (37.2)	77 (34.8)	307 (37.9)	0.458	319 (50.3)	64 (54.2)	0.495
FTC/TDF	459 (44.5)	96 (43.4)	363 (44.8)	0.784	367 (57.9)	90 (76.3)	<0.001
FTC/TAF	149 (14.4)	22 (10.0)	127 (15.7)	0.042	131 (20.7)	18 (15.3)	0.220
PI	271 (26.3)	79 (35.7)	192 (23.7)	<0.001	202 (31.9)	66 (55.9)	<0.001
INSTI	475 (46.0)	82 (37.1)	393 (48.5)	0.003	407 (64.2)	67 (56.8)	0.153
NNRTI	340 (32.9)	82 (37.1)	258 (31.8)	0.161	270 (42.6)	67 (56.8)	0.006
Reasons for switching, n (%)							
Simplification	168 (16.3)	45 (20.4)	123 (15.2)	0.080	119 (18.8)	48 (40.7)	<0.001
Toxicity	61 (5.9)	22 (10.0)	39 (4.8)	0.007	48 (7.6)	13 (11.0)	0.282
Transition therapy to injectable drugs	587 (56.9)	105 (47.5)	482 (59.4)	0.002	493 (77.8)	93 (78.8)	0.895
Drug interaction	9 (0.9)	1 (0.5)	8 (1.0)	0.727	9 (1.4)	0 (0.0)	0.400
Simplicity	33 (3.2)	5 (2.3)	28 (3.5)	0.499	24 (3.8)	8 (6.8)	0.218
Cost	26 (2.5)	5 (2.3)	21 (2.6)	0.974	13 (2.1)	13 (11.0)	<0.001
Coinfections, n (%)							
HBV diagnosis	192 (27.9)	36 (27.1)	156 (28.1)	0.904	139 (24.3)	51 (44.0)	<0.001
HBsAg positive	10 (5.3)	1 (2.8)	9 (5.9)	0.738	4 (2.9)	6 (11.8)	0.043
HCV positive ELISA	160 (23.0)	41 (30.4)	119 (21.2)	0.032	111 (19.2)	49 (42.2)	<0.001
HCV positive PCR	52 (34.7)	16 (42.1)	36 (32.1)	0.359	26 (25.5)	26 (54.2)	0.001
Viral load < 50 copies/mL, n (%)							
Baseline	943 (96.0)	206 (95.4)	737 (96.2)	0.716	576 (95.8)	107 (96.4)	0.991
24 weeks	889 (96.6)	196 (97.5)	693 (96.4)	0.574	532 (96.7)	100 (95.2)	0.638
48 weeks	743 (97.5)	162 (96.4)	581 (97.8)	0.463	393 (97.3)	81 (94.2)	0.258
96 weeks	417 (98.3)	98 (97.0)	319 (98.8)	0.456	126 (97.7)	38 (95.0)	0.735

^ Only patients with data on the absence of AIDS status were included. ^^ In some patients, previous treatments included PI in combination con INSTI. 3TC: lamivudine; AIDS: acquired immunodeficiency syndrome; bPI: boosted protease inhibitor; DTG: dolutegravir, ELISA: enzyme-linked immunosorbent assay; FTC: emtricitabine; HBsAg: hepatitis B surface antigen; HBV: hepatitis B virus; HCV: hepatitis C virus; INSTI: INSTI: integrase strand transfer inhibitor; NNRTI: non-nucleoside reverse transcriptase inhibitor; PCR: polymerase chain reaction; RPV: rilpivirine; TAF: tenofovir alafenamide; TDF: tenofovir disoproxil fumarate.

**Table 2:** The odds ratio of the proposed models associated with nomograms at 24 weeks in PLWHIV to be treated DTG + 3TC.

Variable	OR	IC 95%	*p*-Value
Model 1. To achieve CD4 ≥ 500 cells/mm^3^ at 24 weeks in PLWHIV to be treated DTG + 3TC and with basal CD4 values < 500 cells/mm^3^ [N = 146]			
5–9 years of HIV diagnosis	0.907	0.223–3.684	0.184
Ten or more years of HIV diagnosis	0.391	0.120–1.275	0.156
Age: > 50 years old	0.371	0.141–0.977	0.045
NNRTI	0.349	0.127–0.959	0.041
AIDS	0.299	0.097–0.918	0.035
Baseline CD4+ ≥ 30%	3.295	0.856–12.675	0.083
Model 2. To achieve CD4 ≥ 30% at 24 weeks in PLWHIV to be treated DTG + 3TC and with basal %CD4 < 30% [N = 209]			
AIDS	0.244	0.055–1.094	0.065
FTC/TDF	0.490	0.232–1.035	0.062
Baseline CD4+ ≥ 500 cells/mm^3^	2.433	0.985–6.006	0.054
Baseline CD4/CD8 ≥ 0.9	3.656	1.309–10.214	0.013
Model 3. To achieve CD8 ≤ 1000 cells/mm^3^ at 24 weeks in PLWHIV to be treated DTG + 3TC and with basal CD8 > 1100 cells/mm^3^ [N = 226]			
INSTI	2.013	0.950–4.265	0.068
Comorbidity	0.487	0.251–0.944	0.033
Baseline CD4/CD8 ratio ≥ 0.9	1.955	0.900–4.247	0.090
Model 4. To achieve CD4/CD8 ≥ 0.9 at 24 weeks in PLWHIV to be treated DTG + 3TC and with basal CD4/CD8 < 0.9 [N = 272]			
NNRTI	2.119	0.906–4.955	0.083
Baseline CD4 ≥ 30%	0.336	0.111–1.019	0.054
Baseline CD8 ≤ 1000 cells/mm^3^	0.326	0.129–0.823	0.018
Model 5. To achieve CD4 < 500 cells/mm^3^ at 24 weeks in PLWHIV to be treated DTG + 3TC with basal CD4 values ≥ 500 cells/mm^3^ [N = 489]			
Age: > 50 years old	2.688	0.995–7.266	0.051
Baseline CD4 ≥ 30%	0.411	0.157–1.077	0.070
Baseline CD8 ≤ 1000 cells/mm^3^	4.212	1.305–13.591	0.016
Model 6. To achieve CD4 < 30% at 24 weeks in PLWHIV to be treated DTG + 3TC and with basal ≥ %CD4 30% [N = 361]			
AIDS	2.300	0.993–5.327	0.052
Baseline CD4 ≥ 500 cells/mm^3^	0.198	0.060–0.653	0.040
Baseline CD4/CD8 ≥ 0.9	0.209	0.104–0.418	<0.001
Model 7. To achieve CD8 > 1000 cells/mm^3^ at 24 weeks in PLWHIV to be treated DTG + 3TC and with basal CD8 ≤ 1100 cells/mm^3^ [N = 358]			
Backbone ABC/3TC	0.309	0.563–1.023	0.059
Baseline CD4 ≥ 30%	1.902	0.881–4.107	0.090
Baseline CD4/CD8 ≥ 0.9	0.309	0.152–0.628	0.001
Model 8. To achieve CD4/CD8 < 0.9 at 24 weeks in PLWHIV to be treated DTG + 3TC and with basal CD4/CD8 < 0.9 [N = 272]			
AIDS	0.331	0.096–1.137	0.079
Baseline CD4 ≥ 30%	2.594	1.267–5.310	0.009
Baseline CD8 ≤ 1000 cells/mm^3^	2.071	1.009–4.252	0.047

Abbreviations: DTG: dolutegravir; 3TC: lamivudine; NNRTI: non-nucleoside reverse transcriptase inhibitor; AIDS: acquired immunodeficiency syndrome; FTC: emtricitabine; TDF: tenofovir disoproxil fumarate.

## Data Availability

Data supporting reported results can be found at Infanta Leonor University Hospital. Data can be accessed on request.

## Appendix A

SPADE Study Group: Hospital Burgos, Burgos, Spain (Luis Buzón, Carolina Navarro, María Fernández, Leticia Sánchez); Hospital de Valladolid, Valladolid, Spain (Carlos Dueñas, Laura Rodríguez, Sara Gutiérrez, Genoveva Zapico); Hospital Universitario Infanta Leonor, Madrid, Spain (Jesús Troya, Guillermo Cuevas), Complejo Hospitalario de Navarra, Pamplona, Spain (Estela Moreno-García); Hospital Álvaro Cunqueiro, Vigo, Spain (Guillermo Pousada); Hospital de Huesca, Huesca, Spain (Miguel Egido); Complejo asistencial de Zamora, Zamora, Spain (Cristina Martín); Hospital de Segovia, Segovia, Spain (Eva Ferreira); Hospital la Princesa, Madrid, Spain (Ignacio Santos, Marta del Rey); Hospital Puerta de Hierro, Madrid, Spain (Sara Fuente, Alberto Díaz); Hospital Río Hortega, Valladolid, Spain (Julia Gómez); Hospital Universitario de Áraba, Vitoria, Spain (Miguel Ángel Moran); Hospital Universitario de Donostia, Donostia, Spain (Josean Iribarren); Hospital Universitario de Salamanca, Salamanca, Spain (Alicia Iglesias); Hospital Marqués de Valdecialla, Santander, Spain (Claudia González), Hospital Virgen de la Salud Toledo, Toledo, Spain (María Antonia Sepúlveda).
